# Field strength dependence of grey matter $R_{2}^{*}$ on venous oxygenation

**DOI:** 10.1016/j.neuroimage.2016.10.004

**Published:** 2017-02-01

**Authors:** Paula L. Croal, Ian D. Driver, Susan T. Francis, Penny A. Gowland

**Affiliations:** Sir Peter Mansfield Imaging Centre, School of Physics and Astronomy, University of Nottingham, Nottingham NG7 2RD, UK

**Keywords:** Calibrated BOLD, fMRI, R2* quantification, Oxygenation, Hyperoxia

## Abstract

The relationship between venous blood oxygenation and change in transverse relaxation rate (Δ$R_{2}^{*}$) plays a key role in calibrated BOLD fMRI. This relationship, defined by the parameter *β***,** has previously been determined using theoretical simulations and experimental measures. However, these earlier studies have been confounded by the change in venous cerebral blood volume (CBV) in response to functional tasks. This study used a double-echo gradient echo EPI scheme in conjunction with a graded isocapnic hyperoxic sequence to assess quantitatively the relationship between the fractional venous blood oxygenation *(1−Y*_v_*)* and transverse relaxation rate of grey matter (Δ$R_{2\,\;\;\;\;GM}^{*}$), without inducing a change in *vCBV*.

The results demonstrate that the relationship between Δ$R_{2}^{*}$ and fractional venous oxygenation at all magnet field strengths studied was adequately described by a linear relationship. The gradient of this relationship did not increase monotonically with field strength, which may be attributed to the relative contributions of intravascular and extravascular signals which will vary with both field strength and blood oxygenation.

## Highlights

• We assess the relationship between grey matter $R_{2}^{*}$ and venous oxygenation.
• Isocapnic hyperoxia prevented confounding changes in cerebral blood volume.
• A linear dependency is an appropriate assumption at 1.5, 3 and 7 T.
• Intravascular/extravascular signal ratios will vary with both B0 and oxygenation.

## 1 Introduction

The blood oxygenation level dependent (BOLD) signal arises from the interplay between cerebral blood flow (CBF) and the cerebral metabolic rate of oxygen (CMRO_2_) consumption. However, the magnitude of the BOLD signal also depends on baseline physiological parameters such as cerebral blood volume (CBV), oxygen extraction fraction (OEF) and haematocrit (Hct) (Kim et al., 2012; Levin et al., 2001; Lin et al., 1998). These parameters can vary for a given individual across repeated scan sessions, between healthy subjects, and in particular across patient groups. This results in variability in the magnitude of the BOLD signal, which can be minimised by using the calibrated BOLD signal to measure changes in the CMRO_2_ non-invasively. The original calibrated BOLD model of Davis et al. (1998) provides a non-invasive method to quantify the fractional change in cerebral metabolic rate of oxygen (CMRO_2_) by using the vasodilatory effect of hypercapnia to normalise the BOLD signal.

Current models of calibrated BOLD depend on the relationship between the apparent transverse relaxation rate ($R_{2}^{*}$) and concentration of deoxyhaemoglobin (*dHb*) in the blood, or fractional venous oxygenation (*1−Y*_*v*_) (Ogawa et al., 1993). The contribution to *R*_2_* from deoxygenated blood (Δ $R_{2}^{*}$) can be described as a function of venous CBV (*vCBV*) and blood concentration of *dHb*,

(1) $$∆R_{2}^{*}\;=\;\mathrm{kvCBV}{\left[\mathrm{dHb}\right]}^{\beta }$$

where [*dHb*] is a function of OEF and Hct (Davis et al., 1998), and *k* is a constant incorporating properties such as vessel geometry, magnet field strength (B_0_) and blood-tissue susceptibility differences.

The parameter *β* has traditionally been predicted from Monte Carlo simulations and analytical models (Boxerman et al., 1995; He et al., 2007; Ogawa et al., 1993; Yablonskiy and Haacke, 1994). This involves modelling the microscopic magnetic field gradients between deoxygenated blood vessels and the surrounding diamagnetic tissue to estimate the resulting enhancement of the $R_{2}^{*}$ decay rate. For the extravascular (EV) compartment, the result depends on whether the system is in the static dephasing regime (larger vessels, *β* →1) or the diffusive dephasing regime (smaller vessels, *β*→2). The vessel size limit where the transition between these regimes occurs reduces as B_0_ increases (Kennan et al., 1994), such that Monte Carlo simulations predict the widely accepted value of *β*=1.5 for a vascular network at 1.5 T, and *β*=1 at 7 T (Boxerman et al., 1995, Ogawa et al., 1993; Yablonskiy and Haacke, 1994). However, the reliability of these results depends on how realistic the model is. In particular, models should also consider the effect of changes in intravascular (IV) signal contributions due to concomitant changes in *vCBV*, for example due to vessel dilation in response to functional activation or hypercapnia, and vessel constriction in response to non-isocapnic hyperoxia. This IV contribution can be significant as the $R_{2}^{*}$ of blood increases quadratically with increasing deoxygenation (Blockley et al., 2012, Li et al., 1998). At fields below 3 T the intravascular signal from the veins represents a significant fraction of the BOLD signal, estimated to be approximately 57% at 1.5 T, 36% at 3 T, and negligible at 7 T (Uludag et al., 2009, van der Zwaag et al., 2009), depending on echo time.

More recently, experimental measurements of *β* have been published (Driver et al., 2010, Rossi et al., 2012, van der Zwaag et al., 2009, Wise et al., 2013), which are in reasonable agreement with model-based predictions. However, while theoretical estimates may not fully account for intravascular (IV) signal contributions, experimental measurements to date have been subject to potential concomitant changes in *vCBV*, arising from vessel dilation in response to functional activation or hypercapnia, or vessel constriction in response to non-isocapnic hyperoxia.

This study aims to measure the relationship between fractional change in concentration of deoxyhaemoglobin in venous blood and grey matter $R_{2}^{*}$, without a confounding change in *vCBV*, using a dynamic isocapnic hyperoxic stimulus at 1.5, 3 and 7 T.

## 2 Material and methods

The study was approved by the University of Nottingham Medical School Ethics Committee and all subjects gave prior informed written consent. Eight healthy volunteers (aged 24–27 years, 5 male) participated in the study. MR data was collected on Philips Achieva systems at three B_0_s, a 1.5 T system with 8-channel SENSE receive coil, a 3 T system with 32-channel SENSE receive coil and a 7 T system with head volume transmit and 32-channel SENSE receive coil.

An isocapnic hyperoxic stimulus was delivered to subjects using a feed-forward, low gas flow system (RespirAct™, Thornhill Research Inc., Toronto, Canada) in which arterial blood gases were controlled by a prospective targeting system consisting of a computerized gas blender and sequential gas delivery breathing circuit. The system uses the method of Slessarev et al. (2007) to target end-tidal PCO_2_ (P_ET_CO_2_) and PO_2_ (P_ET_O_2_) independently of each other and independent of breathing pattern (Fierstra et al., 2013). Subjects received medical air via the RespirAct™ until the respiratory challenge commenced. The respiratory paradigm consisted of three minutes of normocapnic (maintaining P_ET_CO_2_ at resting levels) normoxia, followed by a 4 minute normocapnic linear increase in P_ET_O_2_ to a maximum level of 500 mmHg (an approximate 8 mmHg increase in P_ET_O_2_ with each breath), a linear decrease in P_ET_O_2_ to return to baseline over 4 minutes while continuing to maintain normocapnia, and a final 1 minute of normocapnic normoxia. The total respiratory paradigm was thus 12 minutes in duration.

Double echo gradient echo EPI (GE-EPI) data were acquired throughout the respiratory paradigm. At all three magnet field strengths, five contiguous axial slices positioned superior to the ventricles were acquired, with an in-plane resolution of 2×2 mm^3^, 3 mm slice thickness, and field of view of 212×188×15 mm^3^, 212×184×15 mm^3^ and 192×192×15 mm^3^ at 1.5, 3 and 7 T respectively. Imaging parameters were TR of 3 s, SENSE factor 2.5, flip angle 90°, and TE_1_/TE_2_ of 25/80 ms, 22/60 ms and 16/47 ms at 1.5, 3 and 7 T respectively. These double echo GE-EPI echo times were selected to optimally fit T_2_* based on the expected grey matter T_2_* value at each B_0_, as reported in Peters et al. (2007). In addition, at normoxia, a series of inversion recovery GE-EPI images were acquired at 10 inversion times (100, 200, 300, 500, 700, 1000, 1300, 1500, 2100 and 2600 ms) with matched geometry to the double echo GE-EPI data, which were fit for longitudinal relaxation time, T_1_. The first echo (TE_1_) images of the double echo GE-EPI datasets were motion corrected using MCFLIRT (FSL, fMRIB, Oxford, UK) and the resulting transforms were applied to the second echo (TE_2_) images. A grey matter (GM) mask was formed by thresholding T_1_ maps at 1.0≤T_1_≤1.3 s, 1.45≤T_1_≤1.75 s, and 1.8≤T_1_≤2.1 s for 1.5, 3 and 7 T respectively. To remove any remaining contributions in the GM mask from cerebral spinal fluid (CSF) and large veins, those voxels with highest and lowest 3% of signal intensity respectively in the second echo (TE_2_) GE-EPI images were removed from the GM mask. $R_{2}^{*}$ maps were then calculated within the GM mask on a voxel-wise basis using

(2) $$R_{2}^{*}\;\;=\;\;\frac{1}{{\mathrm{TE}}_{2}-{\mathrm{TE}}_{1}}\ln\left(\frac{S_{1}}{S_{2}}\right)$$

where *S*_*1*_ and *S*_*2*_ denote the signals measured at TE_1_ and TE_2_. The mean baseline $R_{2}^{*}$ was then estimated for the GM mask from the normoxic period, and the baseline $R_{2}^{*}$ subtracted from each $R_{2}^{*}$ value on hyperoxia, to obtain a timeseries of Δ$R_{2\,\;\;GM}^{*}$.

The P_ET_O_2_ trace was linearly interpolated to a TR of 3 s and P_ET_O_2_ measurements were converted to venous oxygenation (*Y*_*v*_), assuming a baseline OEF of 0.4 (Chiarelli et al., 2007). *Y*_*v*_ values were binned across the full theoretical range of 0.6 to 0.7, in steps of 0.025 to enable the corresponding $R_{2\,\;\;\;\;GM}^{*}$ data to be group averaged over these bins. For each individual subjects’ data, at each B_0_, the average value of Δ$R_{2\,\;\;\;GM\,\;}^{*}$ within the GM mask was fitted to two models. First, a simple linear function

(3) $$∆R_{2\mathrm{GM}}^{*}\;=\;A\left(1-Y_{v}\right)+C$$

where *(1−Y*_*v*_*) is* proportional to [*dHb*] in Eq. (1), and *A* and *C* are the fitted gradient and intercept (which takes account of the fact that we do not measure Δ*R*_2_*_GM_ with respect to *Y*_*v*_=0). Second, to a power law function

(4) $$∆R_{2\;\mathrm{GM}}^{*}\;=\;A^{\prime }{(1-Y_{v})}^{\beta }+C^{\prime }$$

to estimate *A*′, β and *C*′, using a non-linear, three parameter least squares fit. Data were also fitted to Eq. (4), fixing *β* to published B_0_-corrected values (1.5, 1.3 and 1 for 1.5 T, 3 T and 7 T respectively (Bulte et al., 2009; Davis et al., 1998; Driver et al., 2012)). The Chi-squared (Χ^2^) was used as a measure of the goodness of fit.

We also simulated the relationship between the EV signal and venous oxygenation by using the known blood deoxyhaemoglobin relaxivity to separate the IV component from the total tissue signal, to explore contributions of any residual IV signal which was not accounted for by removal of voxels containing large vessels. While the intravascular relaxation rate $R_{2\,\;\;\;\;IV}^{*}$ varies quadratically with (1-*Y*_*v*_) (Blockley et al., 2008, Li et al., 1998) a linear relationship was assumed across the range of venous oxygenation targeted in this study (0.6–0.7) (Blockley et al., 2008). The resulting estimates of $R_{2\,\;\;IV}^{*}$ were combined with the measured values of Δ $R_{2\,\;\;GM}^{*}$ to estimate the variations in *Δ* $R_{2\,\;EV}^{*}$. First a simple two compartment model was assumed:

(5) $$S_{voxel}\;=\;M_{0}\left(f_{v}.e^{-TE.R_{2\;IV}^{*}}+(1-f_{v}).e^{-TE.R_{2\;EV}^{*}}\right)$$

where *S*_*voxel*_ is the GM signal measured from a voxel at echo time *TE*_*,*_ *ƒ*_*v*_ is intravascular blood volume fraction (assumed to be 3% (Helenius et al., 2003; Lee et al., 2001)) and $R_{2\,\;\;IV}^{*}$ and $R_{2\,\;\;\;EV}^{*}$ correspond to the $R_{2}^{*}$ of the IV and EV compartments respectively, neglecting vessel orientation effects. The ratio of the signal measured at echo times *TE*_*1*_ and *TE*_*2*_ can then be written as:

(6) $$\frac{S_{1}}{S_{2}}\;=\;\frac{f_{v}.e^{-TE_{1}.R_{2\;IV}^{*}}+(1-f_{v}).e^{-TE_{1}.R_{2\;EV}^{*}}}{f_{v}.e^{-TE_{2}.R_{2\;IV}^{*}}+(1-f_{v}).e^{-TE_{2}.R_{2\;EV}^{*}}},$$

Assuming that Δ$R_{2\,\;EV}^{*}$ also varied linearly with Y over the range of interest and a literature value of f, the variation in *S*_*1*_*/S*_*2*_ versus (*1−Y*_*v*_) was fitted to determine the expected values of Δ$R_{2\,\;EV}^{*}$. In this simulation the differences in T_1_ and spin density between tissue and blood were assumed negligible; in fact tissue possesses a slightly lower spin density and shorter T_1_ than blood so these effects will work in opposing directions.

## 3 Results

**Fig. 1:**
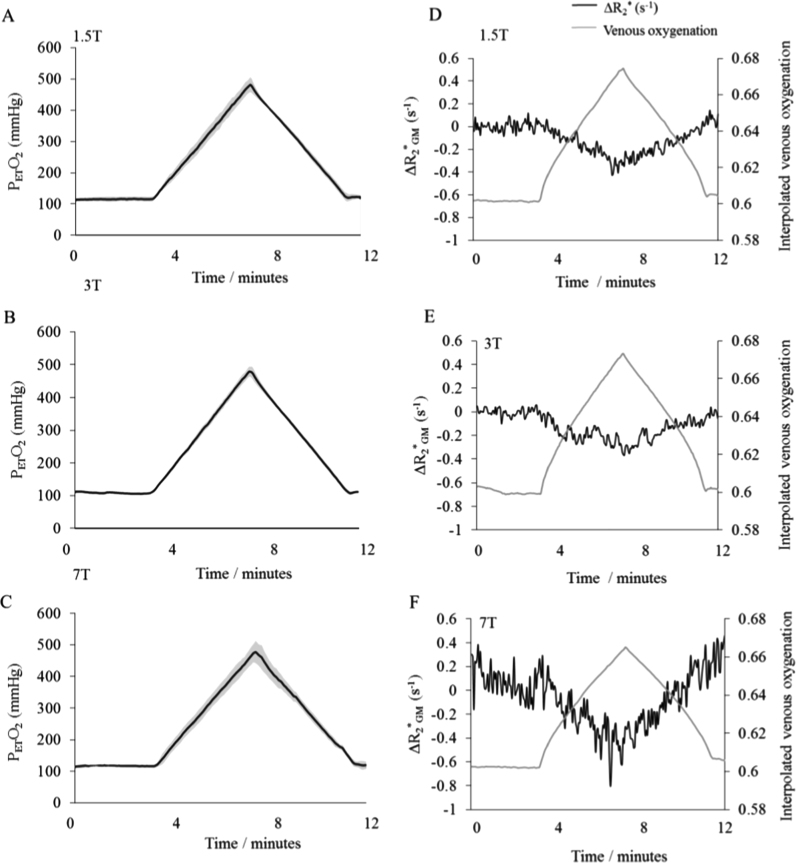
(A–C) Group averages of end tidal partial pressure of oxygen (P_ET_O_2_) shown in black at each field strength across the twelve minute respiratory paradigm. Grey regions indicate standard deviation. (D–F) Interpolated venous oxygenation (Y_v_) time course for representative subjects and accompanying ΔR^*^_2_ (s^−1^) at B) 1.5 T, C) 3 T and D) 7 T.

**Fig. 2:**
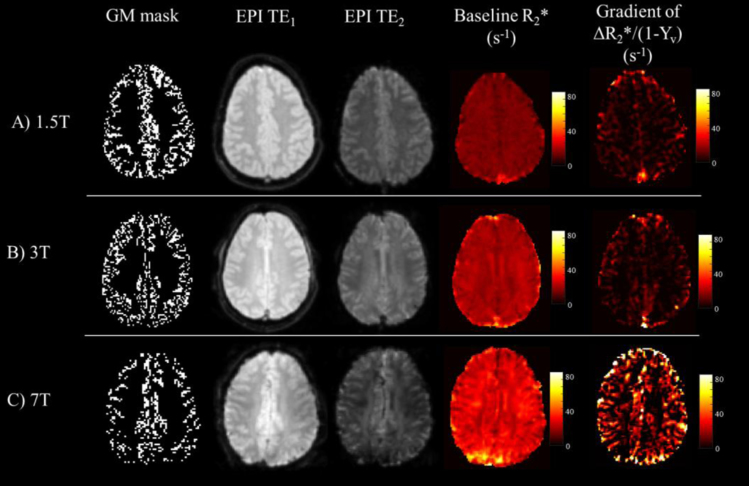
Grey matter (GM) masks, EPIs for TE_1_ and TE_2_ at normoxic baseline, with corresponding R^*^_2_ images, and voxelwise gradient of ΔR^*^_2__GM_ versus *(1−Y*_*v*_*)*. Data shown for representative subjects at A) 1.5 T, B) 3 T and C) 7 T.

Average baseline and peak P_ET_O_2_ values achieved at each B_0_ are shown in Table 1, along with the corresponding *Y*_*v*_ value. Group averaged P_ET_O_2_ traces are shown in Fig. 1A–C. P_ET_CO_2_ values were maintained within ±0.39 mmHg of an individuals’ resting level, with the mean change between baseline normoxia and peak hyperoxia being 0.70±0.12 mmHg, across all subjects and B_0_. Such fluctuations in P_ET_CO_2_ were assumed to have minimal influence on $R_{2}^{*}$. Baseline (normoxic) $R_{2\,\;GM}^{*}$ increased significantly with B_0_ (p < 0.005, Wilcoxon signed rank test). Example *Y*_*v*_ and Δ$R_{2\,\;\;\,GM}^{*}$ timecourses are shown for representative subjects across B_0_ in Fig. 1D–F. Corresponding maps of $R_{2}^{*}$ and the gradient (A) of the linear fit to Eq. (3) are depicted in Fig. 2.

**Fig. 3:**
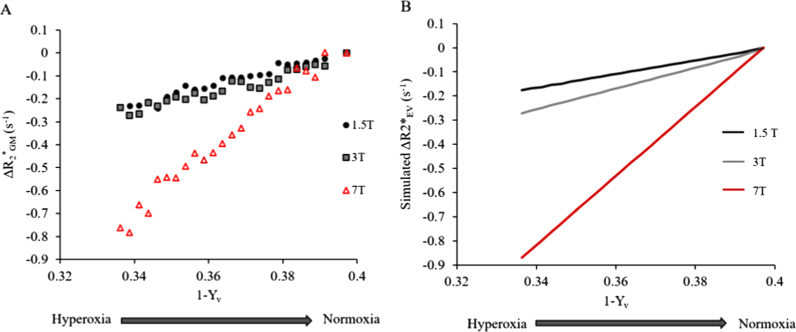
(A) Effect of change in fractional venous oxygenation (1−Y_v_) on A) ΔR^*^_2__GM_ (s^−1^) and B) ΔR^*^_2__EV_ (s^−1^), shown at 1.5 T (black), 3 T (grey) and 7 T (red). Data binned and averaged across subjects. (For interpretation of the references to color in this figure legend, the reader is referred to the web version of this article.)

Fig. 3A shows the effect of the fractional change in venous oxygenation (1−Y_v_) on Δ$R_{2\,\;GM}^{*}$ for each B_0_. The average gradient, A, of the linear fit of Δ$R_{2\,\;\;\;GM}^{*}$ versus (1−Y_v_) (Eq. (3)) is given in Table 1. This was significantly larger for 7 T than 1.5 or 3 T (p=0.043, Kruskall-Wallis one-way ANOVA), although no significant difference was observed between 1.5 and 3 T.

The data was sufficiently described by the linear 2 parameter (A and C) model fit (Eq. (3)) (p<0.01 in all cases). The 3 parameter model, showed no significant improvements in goodness-of-fit (Χ^2^ Difference test) when compared to either of the 2 parameter fits (linear, and fixed B_0_-corrected values of *β*).

**Fig. 4:**
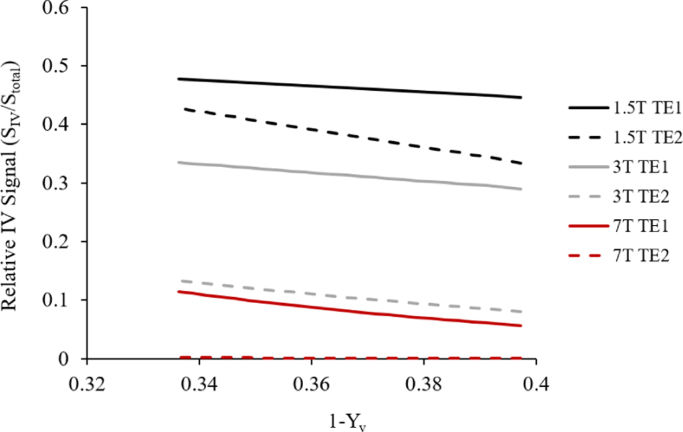
Variation in relative IV signal contribution with (1−Y_v_) estimated for each echo time and field strength.

When $R_{2\,\;\;\;EV}^{*}$ was estimated across binned *Y*_v_ values based on the measured signals and estimated $R_{2\,\;\;\;\;IV}^{*}$, the results demonstrated a monotonic increase in the gradient of Δ $R_{2\,\;\;EV}^{*}$ and (*1−Y*_*v*_) with B_0_ (Fig. 3B). Fig. 4 illustrates subsequent intravascular and extravascular GM signal components for the echo times and field strengths used in this study, for both normoxia and hyperoxia, using Eq. (5).

## 4 Discussion

The relationship between Δ$R_{2\,\;\;\;GM}^{*}$ and fractional venous oxygenation (*1−Y*_*v*_) was measured at 1.5, 3 and 7 T, and found to be well-described by a linear relationship at all B_0_s. This contrasts with previous studies which inferred that *β≥*1 by manipulating B_0_ (Driver et al., 2010, Rossi et al., 2012, van der Zwaag et al., 2009), and by the direct measurement of a value of *β* of 1.35 at 3 T using the response to combined hyperoxia and hypercapnia (Wise et al., 2013). In our study *β* was measured using an isocapnic hyperoxic stimulus and so the results are not confounded by changes in *vCBV* which will have occurred in previous studies, with *vCBV* altered by motor activation (van der Zwaag et al., 2009), the vasodilative effect of hypercapnia (Driver et al., 2010, Wise et al., 2013) or non-isocapnic hyperoxia (Rossi et al., 2012).

Baseline $R_{2\,\;\;GM}^{*}$ increased monotonically and approximately linearly with B_0_, in agreement with published cross-field values (Rossi et al., 2012, van der Zwaag et al., 2009). In contrast, the gradient of Δ$R_{2\,\;\;GM}^{*}$ versus (*1−Y*_*v*_) measured in this study did not increase monotonically (Fig. 3). Extravascular dephasing depends on the intravascular frequency shift (Δ*ω*), which depends linearly on both (*1−Y*_*v*_) and *B*_*0*_:

(7) $$\Delta \omega \;=\;k^{\prime \prime }.\gamma .B_{0}.\Delta \chi .(1-Y_{v})$$

where $k^{\prime \prime }$ is a constant reflecting vessel geometry, *γ* is the gyromagnetic ratio and Δ*χ* is the difference in magnetic susceptibility between fully deoxygenated blood and tissue. Therefore, if we were only sensitive to extravascular signal in the static or indeed diffusive regimes, the gradient of Δ$R_{2\,\;\;GM}^{*}$ versus (*1−Y*_*v*_) would be expected to increase monotonically with B_0_ (Boxerman et al., 1995, Ogawa et al., 1993; Yablonskiy and Haacke, 1994). In practice, the gradient was effectively constant between 1.5 and 3 T and increased faster than linearly with B_0_ between 3 and 7 T (Table 1).

In practice, the non-negligible intravascular (IV) signal will contribute to the signal from GM (Hare et al., 2016), even when signal from large vessels has been masked out, as was the case in this work. Therefore, the unexpected variation in the gradient of Δ$R_{2\,\;\;GM}^{*}$ versus (*1−Y*_*v*_) with B_0_ might be explained by a variation in the ratio of IV signal to EV signal contributions with both B_0_ and echo time (*TE*). Here we combined measured values of Δ$R_{2\,\;GM}^{*}$ with previously published values of $R_{2\,\;\;IV}^{*}$ to estimate Δ$R_{2\,\;\;\;EV}^{*}$, and the results indicated that the gradient of Δ$R_{2\,\;\;\;EV}^{*}$ versus (*1−Y*_*v*_) increased monotonically with B_0_ as expected (Boxerman et al., 1995, Ogawa et al., 1993; Yablonskiy and Haacke, 1994).

If a multi-compartment signal decay is fitted to a single exponential, the apparent $R_{2}^{*}$ will depend on the choice of echo times at which the signal is sampled. Fig. 4 illustrates how the estimated relative contribution of intravascular signal to total signal varies with oxygenation and field strength for the T_2_* values measured here. The IV signal is smaller at normoxia than at hyperoxia, and therefore contributes less to the total signal as (*1−Y*_*v*_) increases. Furthermore, for the echo times used here, the IV signal is more sensitive to changes in fractional venous oxygenation than the EV signal, particularly at lower field. This varying intravascaular signal contribution may explain the unexpected variation of the gradient of Δ$R_{2\,\;GM}^{*}$ versus (*1−Y*_*v*_) with B_0_. However, it should also be noted that any trends in the gradient of a linear fit over a limited range of (*1−Y*_*v*_) could be hard to interpret if the underlying function was not in fact linear. Future studies designed specifically to explore these relationships should use a multi-echo EPI readout and vascular crushing to separate the different IV and EV compartments. In this study the frequency offset (Δ*ω)* between tissue and blood was varied by changing both venous oxygenation and B_0_. To fully characterise the dependency of grey matter*R*_2_* on Δ*ω*, a larger intravascular susceptibility perturbation is required, either by modulating venous blood oxygenation over a wider range, which is difficult to achieve physiologically, or by using a paramagnetic contrast agent.

In summary if the underlying physical value of *β* for any compartment is non-linear, then the effective value of *β* will depend on experimental parameters and local tissue microvasculature (Griffeth et al., 2011; Martindale et al., 2008), but for calibrated BOLD an additional experiment to determine *β* would be impractical. However for the experimental protocol used here, which was deliberately characteristic of standard fMRI experiments, a linear approximation was sufficient to describe the observed behaviour across the limited range of (*1−Y*_*v*_)=0.3–0.4 relevant to the fMRI BOLD effect at all field strengths, which could simplify the calibration of the BOLD signal (Blockley et al., 2015, Griffeth et al., 2013, Merola et al., 2016). Recent work (Griffeth et al., 2011; Merola et al., 2016) has tested calibrated BOLD models by comparing the relatively simple BOLD signal models with a more detailed, multi-compartment BOLD signal model, treating this more detailed model as a gold standard from which to test the specificity of the calibrated BOLD approach to simulated data. The main conclusion of this work was that the physical meaning of the parameter *β* (alongside a physiological CBV/CBF coupling parameter) should be relaxed and their values treated as free parameters.

It has previously been shown that a strong hyperoxic stimulus (FiO_2_=1) will cause macroscopic alterations in inferior brain regions at 3 T which can increase the measured $R_{2\,\;\;}^{*}$, although such effects were reduced under moderate hyperoxia (FiO_2_=0.5) (Pilkinton et al., 2011). Similarly at 7 T, we have previously observed whole-head field shifts under moderate hyperoxia (~FiO_2_=0.6, P_ET_O_2_=500 mmHg), although this only resulted in significant changes in $R_{2}^{*}$ in regions close to the frontal sinus (Driver et al., 2011). In this work such macroscopic effects were mitigated by selecting slices superior to the ventricles, and using moderate hyperoxia. Nonetheless, further quantification of macroscopic inhomogeneities caused by hyperoxia and their impact on $R_{2}^{*}$ are required to enable whole-head analysis. Furthermore, a different paradigm (e.g. sinusoidal (Blockley et al., 2010)) would make it easier to remove any drifts from the data.

## 5 Conclusions

This work presents the first experimental measurement of *β* in humans using an isocapnic hyperoxic stimulus to remove confounds associated with a change in CBV arising from functional activation or modulation of P_ET_O_2_ during a respiratory challenge. It was also found that for the regimes relevant to calibrated BOLD experiments, a value of *β=*1 is a reasonable assumption, which may help simplify the calibrated BOLD model.

## Figures and Tables

**Table 1:** Average baseline and peak P_ET_O_2_ values and corresponding venous oxygenation (Y_v_) estimated according to the interpolation method outlined in Chiarelli et al. (2007). Baseline $R_{2\,\;GM}^{*}(S^{-1})$ and the gradients (A) of Δ$R_{2\,\;GM}^{*}$ versus (1−Y_v_) for both β=1 (linear model) and typical B_0_-adjusted values of *β*. Χ^2^ depicts goodness-of-fit. Data (mean±SEM) averaged across subjects for each magnet field strength (B_0_).

**B**_**0**_**(T)**	**P**_**ET**_**O**_**2**_**(mmHg)**		**Venous oxygenation (Y**_**v**_**)**		**MR parameters for GM mask**			
	Baseline	Peak	Baseline	Peak	Baseline R^*^_2__*GM*_ (s^−1^)	Gradient (*A)* of ΔR^*^_2__*GM*_ v. *(1−Y*_*v*_*)* linear fit (Eq. (3)) (s^−1^)	Χ^2^ for nonlinear fit using B_0_-corrected *β* values (Eq. (4))	Χ^2^ for nonlinear 3 parameter fit (Eq. (4))
**1.5**	115±3	482±11	0.600±0.001	0.670±0.002	12.7±0.2	4.1±0.4 (χ^2^=6.4±2.0†)	χ^2^=6.4±2.0†	χ^2^=6. 4±2.0†
**3**	109±3	485±9	0.600±0.001	0.680±0.001	19.5±0.5	3.9±0.3 (χ^2^=4.3±1.1†)	χ^2^=4.3±1.1†	χ^2^=4.2±1.8†
**7**	119±2	490±13	0.600±0.001	0.670±0.003	33.2±0.7	13.2±2.0 (χ^2^=4.7±1.8†)	χ^2^=4.7±1.8†	χ^2^=4.6±1.8†

† p<0.01.
